# The Structure, Pathogenesis, and Inhibition of the p53-MDM2 Pathway

**DOI:** 10.3390/cancers18040546

**Published:** 2026-02-07

**Authors:** Amanda L. Brown, Xiaoying Lian, Qian Wang

**Affiliations:** 1 School of Medicine, University of South Carolina, Columbia, SC 29209, USA; amanda.brown@uscmed.sc.edu; 2 Department of Chemistry and Biochemistry, University of South Carolina, Columbia, SC 29208, USA; xl27@email.sc.edu

**Keywords:** p53, MDM2 RING domain, MDM2 inhibition, targeted therapy, non-covalent inhibition, rhein-derived compounds

## Abstract

The p53 tumor suppressor protein plays a central role in maintaining genomic stability by regulating cell cycle arrest, apoptosis, and DNA repair under cellular stress. Mouse double minute 2 (MDM2), an E3 ubiquitin ligase, negatively regulates p53 via direct binding and proteasomal degradation. Overexpression or amplification of MDM2 can disrupt this pathway and promote tumorigenesis, even in cancers with wild-type p53. This review outlines the structural features of MDM2, particularly its *N*-terminal hydrophobic pocket and *C*-terminal RING domain, and their roles in p53 regulation. We further examine the pathological effects of MDM2 dysregulation and SNPs linked to increased cancer risk. Recent progress in small molecule MDM2 inhibitors is discussed, with a focus on non-covalent agents such as rhein-derived anthraquinone analogs, including AQ-101, which demonstrate promising anti-cancer activity with reduced toxicity. These findings support the continued development of non-covalent MDM2 inhibitors as a novel therapeutic approach for cancers involving both wild-type and mutant p53.

## Simple Summary

Dysregulation of the p53 tumor suppressor protein has been shown to be a common mechanism underlying the pathogenesis of various types of cancers. A major protein involved in p53 disruption and degradation is the negative regulator MDM2. Several compounds targeting the p53-MDM2 pathway have been developed as promising anti-cancer therapies. Most of these strategies have acted against the p53-MDM2 binding interaction; however, recent research has turned toward inhibiting the MDM2 RING domain. In this review, we outline the structural features of the MDM2 protein, summarize its role in cancer pathogenesis, and review current and promising therapeutic strategies for targeting the p53-MDM2 pathway.

## 1. Introduction

p53 is a tumor suppressor protein that is crucial in coordinating a cell’s response following DNA damage or cellular stress. The important nature of p53 for regulating cellular growth is highlighted by the fact that many cancers, of which uncontrolled cell growth is a hallmark, are associated with mutated or dysregulated p53 [1]. p53 acts as a transcription factor for various genes whose products are responsible for inhibiting cell proliferation in response to DNA damage, either by halting the cell cycle, prompting cellular senescence, or initiating apoptosis [2]. If translocated out of the nucleus and to the mitochondria, p53 can also act as a transcription-independent catalyst for mitochondrial apoptosis [3]. Critical to p53’s function is its ability to induce expression of proteins that modify its own activity via positive or negative feedback. One such protein, mouse double minute 2 (MDM2), is vital to many of p53’s negative feedback loops by operating primarily as an E3 ubiquitin ligase to mark p53 for proteasomal degradation [1,4,5]. While other proteins have been found to play a role in p53 regulation, such as the E3 ligases Cop1 and Pirh-1, these are thought to act more as alternatives in the case of MDM2 deficiency or absence [2,6]. As a result, MDM2 has become a major protein of interest in regard to its ability to modulate p53 levels. In this short review, first, we will discuss MDM2’s structure and function, particularly in relation to its molecular interactions with the tumor suppressor protein p53. We will then examine how changes in MDM2 concentration and activity impact p53 levels and the cellular stress response, focusing largely on MDM2’s role in various cancers. Finally, we will investigate mechanisms of MDM2 inhibition and current known inhibitors, with a focus on the novel method of non-covalent inhibition of MDM2’s RING finger domain.

## 2. General Structure and Function of the p53-MDM2 Complex

**Figure 1:**
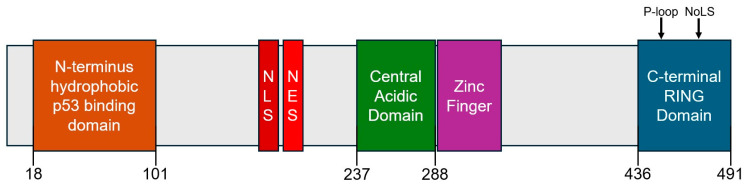
Illustration of different functional domains of mouse double minute 2 (MDM2) protein. From left to right, these include the *N*-terminus p53 binding domain, a nuclear localization sequence (NLS), a nuclear export sequence (NES), the central acidic domain, a Zinc finger domain, and the *C*-terminus RING domain containing a phosphate-binding loop (P-loop) and nucleolar localization sequence (NoLS). Domains of particular importance, including the *N*-terminus p53 binding domain (in orange), the central acidic domain (in green), and the *C*-terminus RING domain (in blue), are provided with their corresponding residue positions.

Initially isolated from a tumorigenic (3T3DM) mouse cell line in 1987, MDM2 is a 489 amino acid protein [7,8]. Its human homolog consists of 491 residues with a predicted molecular mass of 56 kDa, though it migrates at ~85–95 kDa on SDS-PAGE due to an extended acidic region that alters electrophoretic mobility [9,10]. The MDM2 protein is composed of several functional domains: an *N*-terminus hydrophobic cleft, a nuclear localization sequence (NLS), a nuclear export sequence (NES), the aforementioned central acidic domain, a Zinc finger domain, and a *C*-terminus RING finger domain containing a phosphate-binding loop and a nucleolar localization sequence (Figure 1) [10]. Two of these domains, the *N*-terminus hydrophobic region and the *C*-terminus RING finger, are vitally important to MDM2’s ability to impose negative feedback on p53.

Experiments on the binding mechanics between MDM2 and p53 have shown that the hydrophobic *N*-terminus of MDM2 is critical for forming a complex with and inhibiting p53 [5,11]. Structural studies reveal that the *N*-terminal domain (residues 18–101) of MDM2 forms an asymmetric hydrophobic cleft that binds an α-helical region of p53’s *N*-terminus [10,12,13,14]. Key residues on p53—Phe19, Trp23, and Leu26—occupy this cleft, which overlaps with p53’s transactivation domain, thereby impairing its transcriptional activity when bound [9,10,12]. MDM2 can thus inhibit p53’s transcriptional function via this binding interface while also targeting it for degradation through its E3 ligase activity. MDM2 also has a second binding site for p53 within the same hydrophobic domain; however, this is usually inaccessible to wild-type p53 due to blockage by disorderly adjacent areas on MDM2 [15]. This hydrophobic *N*-terminus region of the MDM2 protein is found to be highly conserved between mice and humans, highlighting the biological importance of this region for binding to p53 [9]. Additionally, the central acidic domain (residues 237–288) of MDM2 has been shown to be a binding site for the core DNA binding region of p53, which helps to stabilize binding and aid in ubiquitination [14,16,17,18].

The *C*-terminus RING finger domain of MDM2 (residues 436–491) provides its E3 ubiquitin ligase ability [10,14,19,20]. As an E3 ubiquitin ligase, the RING finger domain catalyzes the formation of peptide bonds between ATP-activated ubiquitin and lysine residues on its target protein. The targets of MDM2 include p53, as well as itself, through auto-ubiquitination, and MDMX (also known as MDM4), a related protein that helps to stabilize MDM2 [19,20]. Similarly to MDM2, MDMX can also bind p53’s *N*-terminus and inhibit its transcriptional ability. However, unlike MDM2, it is not an E3 ubiquitin ligase [19]. MDMX helps to stabilize MDM2 through the formation of MDM2/MDMX heterodimers using their RING domains. These heterodimers favor the ubiquitination of p53 and diminish the autoubiquitination of MDM2, thereby enhancing MDM2’s stability and ability to prompt p53 degradation [19]. Of note, the ratio of MDM2 vs. MDMX determines whether MDMX will act to degrade p53 or sustain it. Low levels of MDMX compared to MDM2 promote p53 degradation through the formation of heterodimers, while comparably high levels of MDMX may form MDMX homodimers, which lack E3 ability and may help to maintain p53 levels by competing against dimers containing MDM2 [19]. Amino acid residues flanking the RING domains of both MDM2 and MDMX are critical to dimerization through the formation of hydrogen bonds, with loss of these *C*-terminal residues severely impacting dimer formation [19]. Residues upstream of the MDM2 RING domain have also been shown to be a potential site of allosteric modification, as phosphorylation of serine residues in this area inhibits RING dimerization and E3 ligase ability [17].

**Figure 2:**
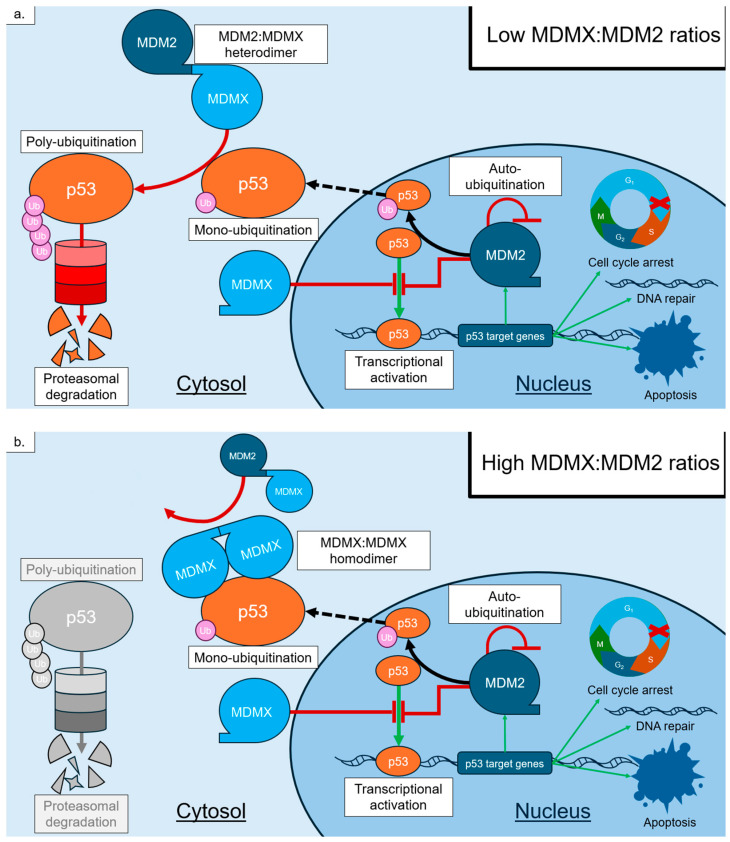
Pathways of p53 modification involving MDM2 and MDMX at varying levels of MDMX. MDM2 is one of the products of p53-mediated transcriptional activation, which is inhibited by MDM2 and MDMX. Mono-ubiquitination of p53 by MDM2 moves it out of the nucleus and to the cytosol, where it can be acted on by MDM2/MDMX heterodimers. (**a**) Low levels of MDMX compared to MDM2 favor the formation of heterodimers leading to proteasomal degradation. (**b**) High levels of MDMX compared to MDM2 result in the formation of MDMX/MDMX homodimers, which block heterodimer-mediated proteasomal degradation and stabilize p53.

As previously stated, MDM2 acts as an E3 ubiquitin ligase for p53, which requires binding to p53’s *N*-terminus transcriptional domain to function. As an E3 ubiquitin ligase, MDM2 can modify p53 functions in several ways depending on the level of ubiquitination. Alone, MDM2 primarily acts to mono-ubiquitinate p53, which stabilizes p53 and facilitates translocation from the nucleus to the cytosol, where it can be further ubiquitinated for degradation or translocated to the mitochondria to induce transcription-independent apoptosis (Figure 2) [1,3,5]. Alternatively, the formation of MDMX-MDM2 heterodimers via their RING domains results in an MDM2 that preferentially poly-ubiquitinates p53 [21]. Poly-ubiquitinated p53 with at least 4 ubiquitin tags on a single lysine residue marks p53 for proteasomal degradation [1,3,5]. This poly-ubiquitination primarily occurs in the cytosol, where MDMX resides in larger quantities, and is dependent on the relative quantities of MDM2 to MDMX, as previously described [21].

While much work has been dedicated to studying the relationship between MDM2 and p53, MDM2 has also been shown to have functions independent of p53, primarily related to cell migration, inducing pluripotency, and maintaining a stem cell phenotype [22,23,24]. In addition to p53, MDM2 has also been shown to target E-cadherin for ubiquitination and degradation via its RING domain [25]. E-cadherin is a transmembrane protein important for cell–cell adhesion, especially in epithelial tissue. In cancer, downregulation or degradation of E-cadherin is a necessary step in the transition of neoplastic epithelial cells to invasive mesenchymal cells that allows for invasion and metastasis [25]. The role that MDM2 may play in cell migration and metastasis is further supported by an increased risk of metastasis in breast cancers with concurrent MDM2 overexpression and E-cadherin down-regulation [25].

Like p53, MDM2 has also been shown to be important to embryonic development. MDM2 is expressed ubiquitously in early embryonic stages, independent of p53 induced expression, and the complete loss of MDM2 in these early stages results in embryonic lethality, largely due to p53 dysregulation [26]. Since p53 is a known regulator of the cell cycle, in vitro removal of it has been utilized in the induction of pluripotent stem cells [22]. MDM2, on the other hand, has been implicated as a contributor to the maintenance of pluripotency [22]. Compared to cells lacking p53 alone, cells deficient in both p53 and MDM2 are significantly less likely to recover pluripotency and show upregulation in several Hox genes associated with cell differentiation [22]. MDM2’s role in inducing pluripotency necessitates an intact and functional RING domain, as mutation of the RING domain has similar outcomes as MDM2-deficient cells [22]. Loss of MDM2 has also been shown to promote stem cell differentiation independent of p53 [22]. MDM2 is capable of physically interacting with and enhancing the function of the Polycomb Repressor Complex 2 (PRC2) protein [22]. PRC2 represses genes associated with differentiation, and therefore overactivation of PRC2 has been implicated in increased aggressiveness in certain cancers, like glioblastoma [27]. These p53-independent functions of MDM2 will not be discussed in detail in this review, but they are mentioned here to further emphasize MDM2, and especially the RING domain, as a promising target for cancer therapy, even in cells where the MDM2-p53 interaction may be altered due to mutated or absent p53.

## 3. The p53-MDM2 Feedback Loop and Pathology

The negative feedback loop that exists between p53 and MDM2 is what allows for the oscillations in p53 levels seen in response to DNA damage [2]. Suppression or inhibition of MDM2 prevents p53 ubiquitination, therefore acting as a primary method to sustain p53 levels in the nucleus [5]. Single-stranded and double-stranded breaks in DNA resulting from IR or UV radiation can lead to the activation of stress-sensitive proteins, like ATR and ATM, which work by stabilizing p53 and degrading MDM2 [2]. The simultaneous activation of p53 and degradation of MDM2 leads to an overall sustained increase in p53 levels, which triggers apoptosis in these damaged cells. Meanwhile, more short-lived bursts of p53 caused by an undisturbed MDM2-p53 feedback loop may temporarily halt cell proliferation but not necessarily lead to apoptosis. p53 can also activate miRNAs to aid in arresting the cell cycle and managing gene transcription. Several of these miRNAs, namely miR-605, miR-143, and miR-145, have been found to play a role in positive feedback loops for p53 via the inhibition of MDM2 transcription and protein synthesis [2].

Due to its importance in regulating the cell cycle in response to stressors, mutations in p53 are strongly linked to uncontrolled cellular growth. In fact, the gene encoding p53, TP53, accounts for the most frequently mutated gene across human cancers at about 35–50%, with particularly high correlations in ovarian serous cystadenocarcinoma, lung squamous cell carcinoma, and small cell lung cancer [28,29]. Germline mutations in p53, as found in Li Fraumeni syndrome, also result in heightened risks for developing cancers, especially leukemia and cancers of the breasts, brain, and adrenal glands [30]. The most frequent TP53 mutations associated with cancers are loss of function mutations that result in reduced transcriptional ability or impaired DNA binding [30]. Alternatively, a reduction in the ability of p53 to regulate the cell cycle can also occur in response to elevated MDM2 levels due to an increase in p53 degradation.

Considering that increases in MDM2 levels promote cell growth by reducing p53 mediated pauses in the cell cycle, pathways such as overexpression or gene amplification of MDM2 provide an alternative mechanism for the development of cancer and other tumors, especially in cases with wild-type p53. Indeed, gene amplification of MDM2 is found in about 7% of tumors, most prominently in soft tissue tumors and osteosarcomas [31]. Overexpression of MDM2 has also been detected in other cancer types, including chronic lymphocytic leukemia, acute lymphoblastic leukemia, and low-grade B-cell lymphoma [32,33]. Furthermore, an observable single-nucleotide polymorphism (SNP) in the promoter of MDM2 (also known as SNP309T > G) has been shown to cause overexpression of MDM2 by increasing its affinity for the transcription factor, Sp1 [34]. This SNP is also associated with accelerated tumor formation and development in Li Fraumeni syndrome, as well as increased risk of various cancers, including leukemia, endometrial cancer, breast cancer, and colorectal cancer [34,35,36,37,38]. Interestingly, the increased cancer risk associated with SNP309T > G is found to be especially strong in Asian populations, potentially due to an overlapping lower incidence of another “cancer-protective” SNP, SNP285C, which reduces Sp1 affinity for MDM2’s promoter [39]. Similarly to MDM2, overexpression of MDMX has also been associated with increased cancer risks, as seen in retinoblastoma, melanoma, and colon cancers [14]. As is evident by the countless studies on the relationship between the p53-MDM2 feedback loop and cancer, inhibition of MDM2 has become a major target for novel cancer therapies due to the potential to indirectly control p53 levels and halt the development of cancerous cells.

## 4. MDM2 Inhibition

As previously stated, MDM2 acts on p53 via two primary mechanisms: either by blocking its *N*-terminal transactivation domain or by marking it for proteasomal degradation. Correspondingly, the development of MDM2 inhibitors has tended to target two main sections, with those being the *N*-terminal binding pocket for p53 and the C-terminal RING domain, which imparts its E3 ligase function.

### 4.1. Targets of MDM2 N-Terminal p53-Binding Domain

**Figure 3:**
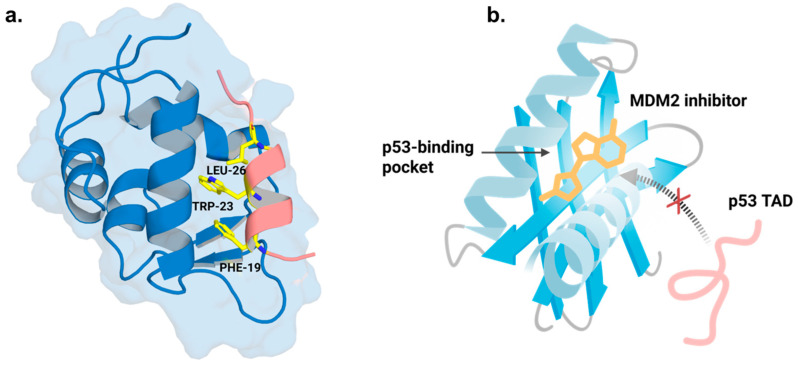
(**a**) Structure of the p53 transactivation domain (TAD, salmon) bound to the N-terminal p53-binding pocket of MDM2 (blue), illustrating the canonical three-finger binding mode (PDB ID: 1YCR; generated using PyMOL 3.1.3). (**b**) Schematic showing how small-molecule MDM2 inhibitors can occupy the p53-binding pocket and competitively block p53 TAD binding, thereby disrupting the MDM2–p53 interaction (Created in BioRender. Lian, X. (2026) https://BioRender.com/vxlvvl1).

A majority of prior efforts at MDM2 inhibition focused on disrupting the p53-MDM2 interaction by creating compounds to mimic the key p53 residues that fit into MDM2’s hydrophobic binding pocket (Figure 3). The first small-molecule inhibitors developed for this purpose were the Nutlins, a class of cis-imidazoline analogs designed to mimic key hydrophobic residues of p53 that occupy the MDM2 binding cleft [40].

**Figure 4:**
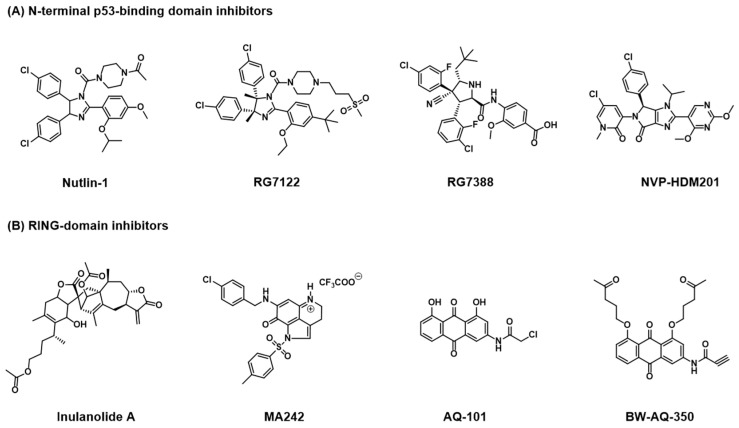
Representative small-molecule inhibitors of MDM2. A comprehensive list of MDM2 inhibitors and their corresponding references is provided in Appendix A, Table A1 and Table A2.

Nutlins are capable of occupying the spaces meant for the key hydrophobic p53 residues, Trp23, Leu26, and Phe19, with two halophenol groups and an ether group, respectively [40]. Despite showing activation of p53 and inhibition of tumor growth in several cancerous cell lines with wild-type p53, these compounds were found to be suboptimal for pharmacological development, resulting in their modification and the creation of RG7112, a modified version of Nutlin 3a and the first MDM2 inhibitor to advance to human clinical trials [23,40,41]. Other small molecules blocking the MDM2 binding pocket have been developed and show similar p53 activation and tumor regression in p53 wild-type cancer cell lines. These include several spiro-oxinidole-based compounds, such as MI-63, MI-888, SAR405838, and APG-115, the pyrrolidine compound RG7388, the spiro-indolinone-based compound RO8994, and the pyrazolopyrrolidinone-based compound NVP-HDM201 (Figure 4) [42,43,44,45,46,47,48,49].

### 4.2. Targets of the MDM2 RING Domain

#### 4.2.1. Covalent Inhibitors

Targeting the MDM2 RING domain has emerged as an alternative strategy to classical disruption of the p53–MDM2 binding interaction. Unlike inhibitors that solely block p53 binding, RING domain–directed approaches aim to directly suppress MDM2 E3 ubiquitin ligase activity, thereby stabilizing p53 and potentially inhibiting p53-independent oncogenic functions of MDM2. Within this category, both covalent and non-covalent inhibitors have been developed, each with distinct mechanistic features and therapeutic implications [50,51]. Covalent inhibition of the MDM2 RING domain represents one of the earliest approaches to directly suppress MDM2 E3 ubiquitin ligase activity. By irreversibly modifying residues within or adjacent to the RING domain, these inhibitors disrupt MDM2-mediated ubiquitination, promote MDM2 autoubiquitination, and interfere with MDM2–MDMX heterodimer formation. As a result, covalent RING domain inhibitors can stabilize p53 and reduce MDM2 protein levels, producing anti-tumor effects that extend beyond tumors with wild-type p53.

One of these small molecules, the dimeric sesquiterpenoid inulanolide A, acts as a MDM2 inhibitor by covalently binding MDM2’s RING domain and blocking the formation of MDM2-MDMX heterodimers [50]. This results in the downregulation of MDM2 via destabilization and auto-ubiquitination [50]. Importantly, whereas small molecular inhibitors targeting the MDM2-p53 binding interaction only showed promise in cells with wild-type p53, inulanolide A showed reduction in cancer cell growth and migration in both wild-type p53 cells and those with mutated or absent p53 [50]. This is noteworthy considering that a significant proportion of cancers bear some form of mutated p53. Another small molecule, the makaluvamine analog MA242, has also been shown to inhibit MDM2 by targeting its RING domain and promoting autoubiquitination [51]. Similarly to inulanolide A, MA242 proved to be effective at selectively inhibiting growth and inducing apoptosis in prostate, breast, and hepatocellular cancer cell lines regardless of p53 status [51,52,53]. Molecular docking studies of these small molecules showed similarities in that they both necessitate the formation of hydrogen bonds with the MDM2 residue Lys446, among others, to support covalent modification [50,52]. Additionally, both of these small molecular inhibitors also show dual function as an inhibitor of the protein NFAT1, which acts as a transcription factor to induce MDM2 expression independent of p53, further downregulating MDM2 levels [50,51,52,53].

#### 4.2.2. Non-Covalent Inhibitors

In contrast to covalent approaches, non-covalent inhibition of the MDM2 RING domain represents a more recent and conceptually distinct strategy aimed at reducing toxicity while retaining therapeutic efficacy. Non-covalent inhibitors rely on reversible interactions, such as hydrogen bonding and hydrophobic contacts, to modulate MDM2 activity, potentially offering improved tolerability compared to irreversible covalent modifiers. There has been recent development of rhein-derived analogs built upon an anthraquinone core that act to non-covalently inhibit MDM2’s RING domain. While an anthraquinone framework is necessitated for the cytotoxic activity of multiple chemotherapeutic drugs such as doxorubicin, it tends to result in undesirable side effects, notably cardiotoxicity [54]. Meanwhile, the naturally derived molecule rhein, despite retaining an anthraquinone framework, offers lower toxicity and higher tolerability in humans while retaining slight anti-tumor potential [55,56].

The first of these rhein-derived small molecules, AQ-101, was originally developed to be a less toxic but equally potent chemotherapeutic agent. However, it was shown to induce MDM2 self-ubiquitination, subsequent proteasomal degradation, and p53 upregulation [55]. This molecule also displayed complete remission of acute lymphoblastic leukemia (ALL) in mice, along with very low toxicity in both mice and ALL cell lines. Furthermore, a lack of time-dependent inhibition when tested in ALL cells indicated that AQ-101 worked as a non-covalent disruptor of MDM2: MDMX heterodimers, despite the presence of a Michael acceptor, suggesting a covalent mechanism [55]. A noncovalent mechanism of action is novel for targeted MDM2 inhibitors, which have up until now tended to rely on covalent inhibition, and may be responsible for the improved tolerability despite the presence of an anthraquinone framework.

Importantly, the reduced toxicity profile of AQ-101 and related rhein-derived analogs may be mechanistically linked to their non-covalent mode of action. Unlike classical anthracycline chemotherapeutics such as doxorubicin, which exert cytotoxic effects through DNA intercalation and inhibition of topoisomerase IIβ—mechanisms strongly associated with cumulative cardiotoxicity—non-covalent MDM2 RING-domain inhibitors primarily target protein–protein interactions rather than DNA [57,58]. Furthermore, the reversible nature of non-covalent binding may limit long-term off-target accumulation and reduce toxicity in cardiomyocytes. Together, these features suggest that non-covalent anthraquinone analogs represent a conceptually distinct class of MDM2 inhibitors that decouple the anthraquinone scaffold from the cardiotoxic mechanisms traditionally associated with this chemical framework.

Due to its low water solubility, AQ-101 lacked the desired pharmacodynamic properties for further development, resulting in the development of the compound BW-AQ-238, along with several amino acid prodrugs, which showed improved solubility and comparable cytotoxicity [59]. Further modification of BW-AQ-238 resulted in the more potent compounds BW-AQ-295 and BW-AQ-350, with BW-AQ-350 showing potency comparable to doxorubicin in wild-type p53 ALL cell lines by downregulating MDM2 [60]. It is important to note that the potency of the mentioned anthraquinone compounds was only found in wild-type p53 cell lines, with inhibition diminishing in cells with mutated or null p53 [55,60]. The exact mechanism of action for the recently discovered compounds BW-AQ-295 and BW-AQ-350 has yet to be elucidated, specifically whether they act in a noncovalent manner similar to their parent compound, AQ-101, or a covalent method. However, the potential development of a class of small molecular inhibitors that act in a non-covalent fashion not only introduces a novel method for MDM2 inhibition but may also result in cancer drugs with reduced toxicity and side effects compared to current options.

## 5. Conclusions

In summary, MDM2 acts as an important negative feedback regulator of p53, which keeps levels low in the absence of other p53 stabilizing proteins. MDM2 accomplishes this negative feedback by either binding to p53’s trans-activation domain and blocking the transcription of downstream tumor suppressor genes or by ubiquitination to remove p53 from the nucleus and mark it for proteasomal degradation. These two mechanisms of p53 regulation are executed by two distinct domains of the MDM2 protein, the *N*-terminal binding domain and the *C*-terminus RING domain, respectively. In addition to negative feedback, MDM2 has also shown p53-independent functions, such as cell migration, inducing pluripotency, and maintaining a stem cell phenotype, all performed by the RING domain. Due to its close relationship with p53, MDM2 overexpression has been implicated in several cancers, especially soft tissue tumors, leukemias, and lymphomas. This association has prompted the creation of various small molecular inhibitors targeting MDM2 as a potential anti-cancer therapy.

The recent development of MDM2 inhibitors proposes an attractive alternative method for the future development of cancer therapeutics, which warrants further study and development. Given the intracellular nature of MDM2, small molecular agents are one of the main methods for inhibition, along with small peptides and antisense oligonucleotides, and offer a promising approach towards novel therapies for certain cancers with MDM2 overexpression or wild-type p53 [23]. While the development of small molecular inhibitors of MDM2 has primarily focused on disrupting the MDM2-p53 binding complex, a few inhibitors have been established to target MDM2’s RING domain and show promising anti-cancer effects even in cells with mutated or absent p53, a previous limitation of molecules directed solely toward the MDM2-p53 complex. We anticipate that the development of small molecular inhibitors of MDM2’s RING domain would theoretically offer a novel and less toxic mechanism for treating cancers with both wild-type and mutated p53.

## Data Availability

The data presented in this study are available on request from the corresponding author.

## Abbreviations

The following abbreviations are used in this manuscript:

MDM2	Mouse double minute 2
SNP	Single-nucleotide polymorphism
ALL	Acute lymphoblastic leukemia

## Appendix A. Summary and Structure of MDM2 Inhibitors

**Table A1:** Summary of inhibitors targeting the N-terminal p53-binding domain of MDM2.

Inhibitor Name	Structure	Reference
Nutlin-1		[35]
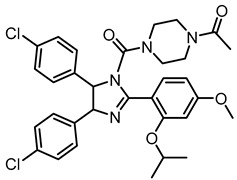
Nutlin-2		[35]
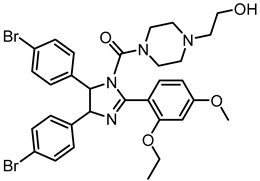
Nutlin-3		[35]
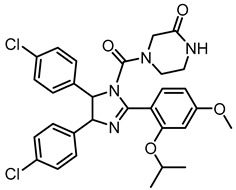
RG7112		[36]
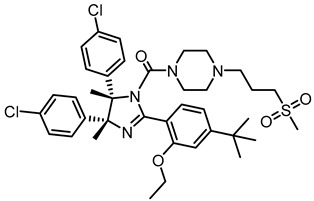
MI-63		[37]
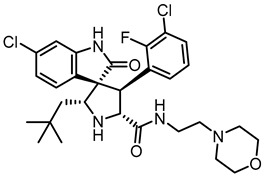
MI-888		[38]
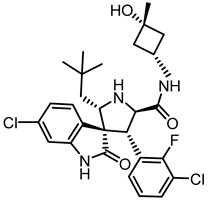
RG7388		[39]
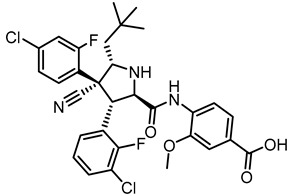
APG-115		[40]
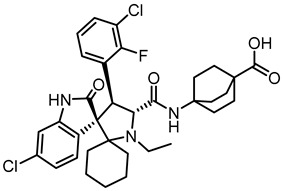
RO8994		[41]
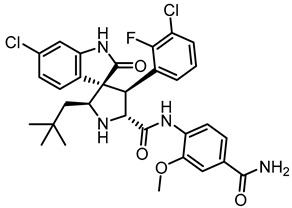
SAR405838		[42]
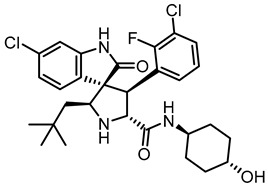
NVP-HDM201		[44]
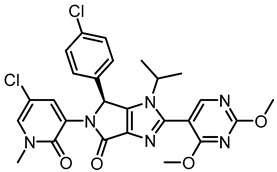

**Table A2:** Summary of inhibitors targeting the RING domain of MDM2.

Inhibitor Name	Structure	Reference
Inulanolide A	****	[45]
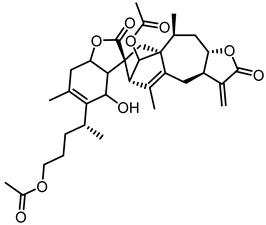
MA242	****	[46]
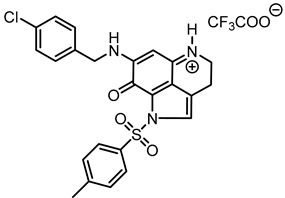
AQ-101	****	[47]
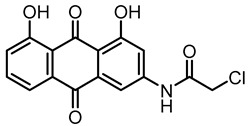
BW-AQ-238	****	[48]
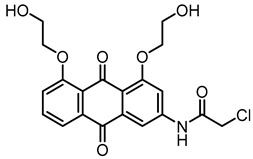
BW-AQ-295	****	[49]
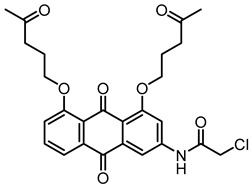
BW-AQ-350	****	[49]
